# Roles of phytohormone changes in the grain yield of rice plants exposed to heat: a review

**DOI:** 10.7717/peerj.7792

**Published:** 2019-11-19

**Authors:** Chao Wu, She Tang, Ganghua Li, Shaohua Wang, Shah Fahad, Yanfeng Ding

**Affiliations:** 1 College of Agronomy, Nanjing Agricultural University, Nanjing, Jiangsu, China; 2 Jiangsu Collaborative Innovation Center for Modern Crop Production, Nanjing, Jiangsu, China; 3 Department of Agronomy, University of Swabi, Swabi Kyber Paktunkhwa, Pakistan

**Keywords:** Rice grain yield, Heat stress, Spikelet fertility, Grain weight, Spikelets per panicle, Phytohormone homeostasis

## Abstract

During its reproductive phase, rice is susceptible to heat stress. Heat events will occur at all stages during the reproductive phase of rice as a result of global warming. Moreover, rice yield traits respond differently to heat stress during panicle initiation, flowering and grain filling. The reduction in the number of spikelets per panicle of heat-stressed plants is due to the attenuated differentiation of secondary branches and their attached florets as well as the promotion of their degradation during the panicle-initiation stage but is not affected by heat stress thereafter. Spikelet sterility as a result of heat stress is attributed not only to physiological abnormalities in the reproductive organs during the flowering stage but also to structural and morphological abnormalities in reproductive organs during the panicle-initiation stage. The reduced grain weight of heat-stressed plants is due to a reduction in nonstructural carbohydrates, undeveloped vascular bundles, and a reduction in glume size during the panicle-initiation stage, while a shortened grain-filling duration, reduced grain-filling rate, and decreased grain width contribute to reduced grain weight during the grain-filling stage. Thus, screening and breeding rice varieties that have comprehensive tolerance to heat stress at all time points during their reproductive stage may be possible to withstand unpredictable heat events in the future. The responses of yield traits to heat stress are regulated by phytohormone levels, which are determined by phytohormone homeostasis. Currently, the biosynthesis and transport of phytohormones are the key processes that determine phytohormone levels in and grain yield of rice under heat stress. Studies on phytohormone homeostatic responses are needed to further reveal the key processes that determine phytohormone levels under heat conditions.

## Introduction

Human activities have triggered climate change, which is manifested as global warming. Global climate change represents one of the most serious challenges faced by humans (Marsicek et al., 2018). Crop production and food security are essential components of agriculture and are severely affected by climate change. Rice is a staple food for approximately half of the global population and frequent heat waves have severely affected rice production (Zhang et al., 2014). An analysis of historical data showed that rice grain yields have decreased by 14% for every 1 °C increase in average daily temperature (Aggarwal, 2009).

Rice plants are vulnerable to heat stress, especially during the reproductive phase (Boote et al., 2005). Heat stress is predicted to occur more frequently and unpredictably as global warming continues to worsen, which may cause rice plants to suffer heat stress at any time during their reproductive stage (Marsicek et al., 2018). Many studies have evaluated how rice plants respond to heat stress and their underlying mechanisms during the mid-late reproductive phase (Wu & Cui, 2014). A risk assessment of the spatiotemporal variation of high-temperature events during the past 20 years indicated that heat events occur in the middle and lower reaches of the Yangtze River, which constitute one of the major paddy rice production areas in China, as early—mid-July (Lin, Wang & Zhang, 2016), during which panicle formation of midseason rice occurs, resulting in disrupted panicle development and ultimately reduced yields (Wu et al., 2016). It was previously reported that rice plants respond differently to heat stress during different developmental stages (Shi et al., 2015). However, few articles provided conclusive evidence concerning the different effects of heat during different reproductive stages on rice grain yields (Fahad et al., 2018). Therefore, the present review provides a comprehensive conclusion of the effects of heat on rice grain yield during the reproductive phase (panicle initiation, flowering and grain filling) and the distinct variations in rice grain yield on the basis of the timing of the heat stress during the three reproductive stages are also identified.

Phytohormones play an important role in coordinating the response of yield traits to heat stress in rice. For example, cytokinin (CTK) and abscisic acid (ABA) modulate floret differentiation and the number of spikelets per panicle and indole-3-acetic acid (IAA) and gibberellin (GA) are thought to be involved in regulating the development of reproductive organs and activities involving pollination and fertilization and thus spikelet fertility. IAA, GA, ABA and CTK mediate grain weight under heat (Wu et al., 2016). However, most previous related studies have explored the phytohormone mechanisms governing the effects of heat stress on rice grain yield by evaluating phytohormone levels (Fahad et al., 2015). Phytohormone levels in target organs are associated with processes involved in phytohormone homeostasis, for example, biosynthesis, catabolism, deactivation and transport (phytohormones acting as mobile signals should be considered) (Sakakibara, 2010), which are purported to be influenced by heat stress, as demonstrated by reports of changes in the activity of enzymes involving phytohormone homeostasis (Wu et al., 2017). The majority of the previous related studies emphasized the role of biosynthesis on the accumulation of target phytohormones (Wani et al., 2016), but studies on the responses of phytohormone homeostasis to heat stress and their roles in rice grain yield are far from adequate. This review provides comprehensive insights into the physiological mechanisms of the heat stress response by considering the roles of phytohormone (CTK, IAA, GAs and ABA) homeostasis on rice grain yield.

## Survey methodology

Research papers published both in English and in Chinese were searched from the Web of Science and the China National Knowledge Infrastructure (the largest Chinese Academic Journals database) from 1950 to 2018. There were six search terms queried as follows: “rice AND high temperature/heat/warming” and “high temperature/heat/warming AND phytohormone”. An initial search resulted in 26,618 articles, which were reduced to 8,813 by limiting research to plant science, agronomy, cell biology, physiology, biology, developmental biology and environmental science. We focused mainly on the physiological and molecular aspects involving phytohormone homeostasis in rice varieties in response to heat stress. We then examined the article titles, abstracts and key words to judge their relevance, after which 2,115 articles were considered relevant. There were 15 studies in other crop species (barley, wheat, tomato, etc.) and in Arabidopsis that were highly relevant to the topic of this review, which were also included.

## Effects of heat stress on grain yield and yield components

### Definition of heat stress and the maximum temperature for rice growth

Heat stress is defined as an increase in temperature beyond a critical threshold and for a certain period of time resulting in irreversible damage to plant growth and development (Wu, 2016). Heat injury depends on the intensity and duration of exposure to high temperature and can be subdivided into two categories: (i) short periods of exposure to extreme high temperatures and (ii) long periods of exposure to sub-high temperatures (Berry & Bjorkman, 1980). Rice plants are highly susceptible to heat stress during their reproductive phase. The early reproductive phase in rice is referred to as the period from panicle initiation to booting (stages R0–R3 according to Counce, Keisling & Mitchell (2000)), during which the maximum temperature for rice growth is 33.1 °C (Sánchez, Rasmussen & Porter, 2014). The mid-late reproductive phase represents the period from heading to physiological maturity and includes both flowering (stage R4 according to Counce, Keisling & Mitchell (2000)) and grain filling (stages R5–R9 according to Counce, Keisling & Mitchell (2000)); the maximum temperature for rice growth is 37 °C and 31.3 °C during flowering and grain filling, respectively (Itoh et al., 2005; Sánchez, Rasmussen & Porter, 2014). Heat stress during the rice reproductive phase reduces grain yield, but the effects on grain yield and its components differ when heat stress occurs during the different reproductive stages (Shi et al., 2015).

### Effect of heat stress on the number of spikelets per panicle

Heat stress reduces the number of spikelets per panicle during the panicle-initiation stage, but the number of spikelets per panicle does not decreased when heat events occur after anthesis (Fahad et al., 2018). The reduction in the number of spikelets per panicle in heat-stressed plants is due to the attenuated differentiation of secondary branches and their attached florets as well as the promotion of their degradation during the panicle-initiation stage, but heat stress does not affect the differentiation of primary branches or their attached florets (Wu et al., 2016). Compared with that of primary branches and their attached florets, the differentiation of secondary branches and their attached florets is more sensitive to environmental factors (Ding et al., 2014).

### Effect of heat stress on spikelet fertility

**Figure 1:**
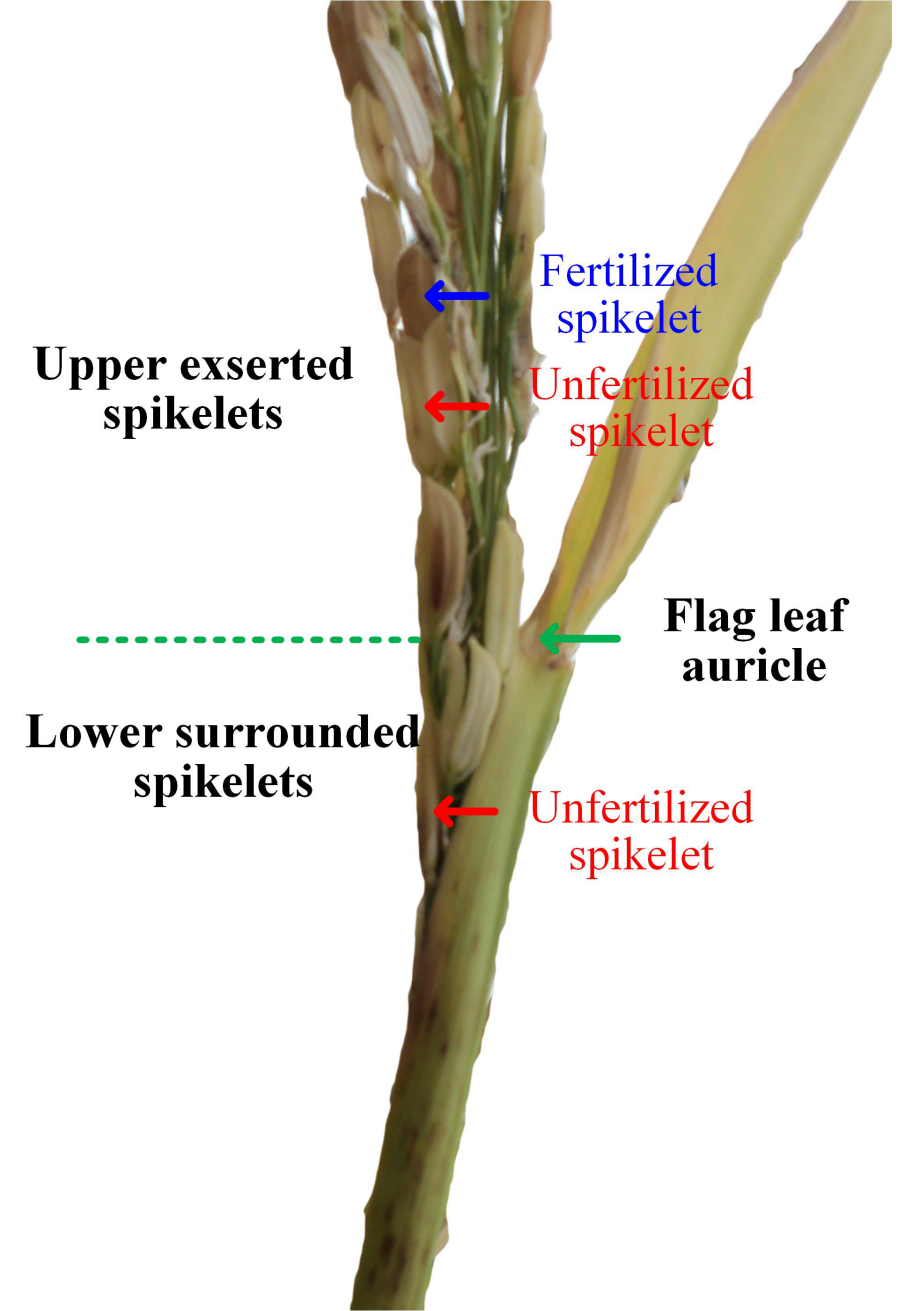
Illustrations of panicle enclosure induced by heat stress (Image credit: Chao Wu).

Heat stress during the early reproductive stage induces panicle enclosure, which manifests as a partial panicle trapped within the flag leaf sheath (Fig. 1; Das et al., 2014; Wu et al., 2016). In the enclosed panicles of heat-stressed plants, the spikelets in the lower region are surrounded by an enclosed sheath (the lower surrounded spikelets), while the spikelets in the upper region of the panicle can successfully expand out of the sheath (the upper expanded spikelets), as illustrated in Fig. 1. With respect to the lower surrounded spikelets, spikelet sterility in heat-stressed plants is associated with failure of pollination due to the physical hindrance of the enclosing sheath (Wu et al., 2016). For the upper expanded spikelets, structural abnormalities in the anthers, disruptions in the function of the septum and tapetum (Wang et al., 2016a), inhibition of microsporogenesis, decreases in pollen sources, reductions in both starch accumulation in and cytoplasm of pollen (Sakata et al., 2000) and morphological and physiological abnormalities of the stigma (Takeoka et al., 1991) collectively reduce the possibility of successful pollination in heat-stressed rice plants (Matsui & Omasa, 2002).

**Figure 2:**
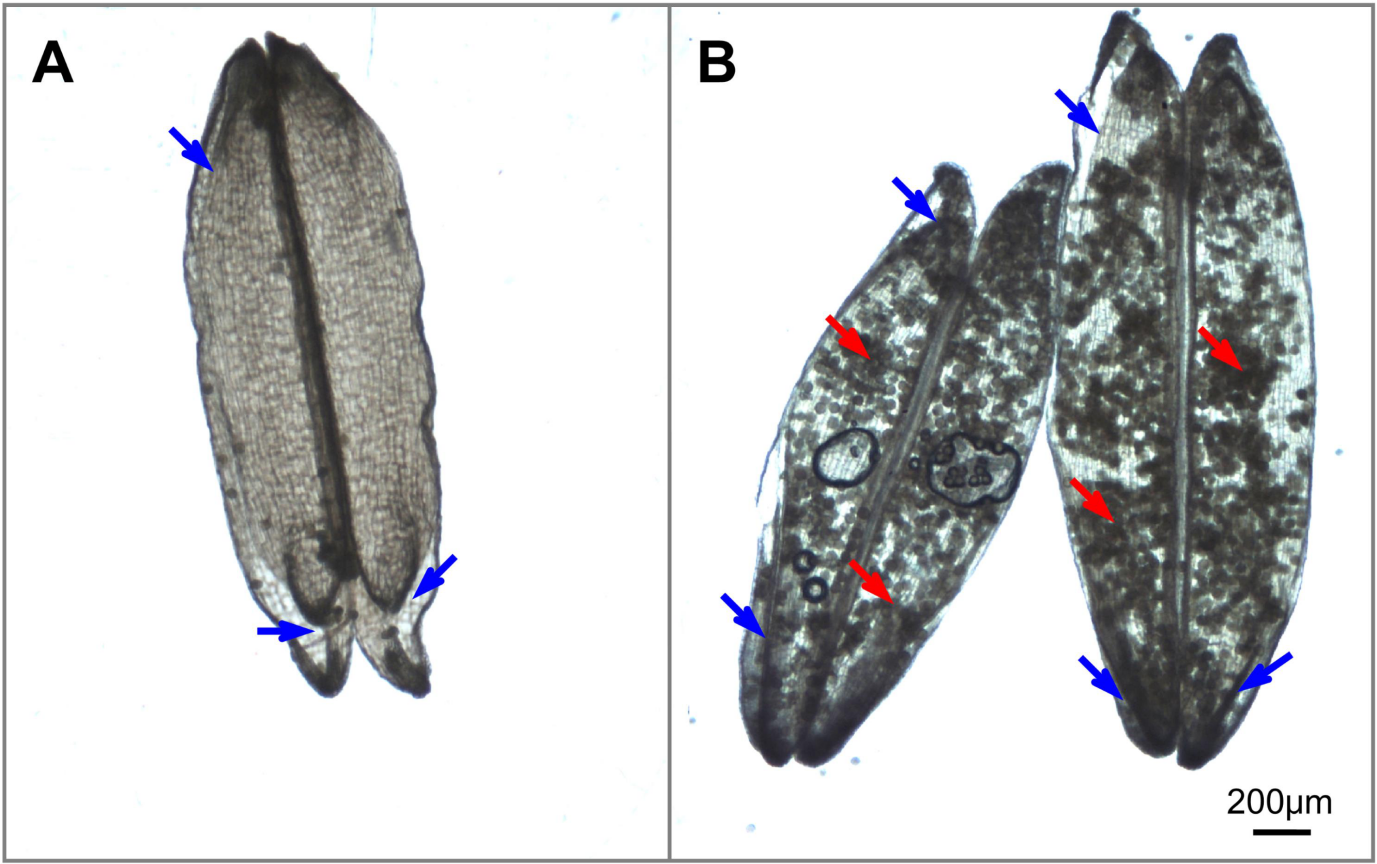
Illustrations of anther dehiscence and pollen release (Image credit: Chao Wu). (A) Dehisced anthers with no adhered pollen under normal temperature. (B) Dehisced anthers with adhered pollens inside the anthers under heat stress. The red arrow indicates residual pollen grains in anthers, and the blue arrow indicates the aperture of the thecae.

During the flowering stage, spikelet sterility in heat-stressed plants is associated with reduced functionality of female and male organs. Impaired female and male organs account for 34% and 66%, respectively, of spikelet sterility under heat stress conditions (Fábián et al., 2019). Four main behaviors of female and male organs affect pollination and fertilization, which are affected by heat stress. (i) The first is inhibition of anther dehiscence. Heat stress delays or even blocks septum ruptures in anthers because of the inhibition of pollen swelling (Wilson et al., 2011), which is partially attributed to a disturbance in water metabolism under heat stress conditions (Matsui & Omasa, 2002). (ii) The second is a reduction in pollen shedding. Heat stress hinders pollen shedding from both indehiscent anthers and dehiscent anthers. It is well known that pollen grains are unable to escape from indehiscent anthers induced by heat stress (Matsui & Omasa, 2002). In dehiscent anthers, heat stress results in the formation of sticky pollen grains that are retained inside the locules by the disruption of anther dehydration, as supported by our previous observations (Fig. 2B). The resultant reduced pollen shedding results in insufficient numbers of pollen grains deposited onto the stigma. (iii) The third is a decrease in stigma receptivity. Heat stress influences stigma peroxidase and stigma-surface esterase activity and thus impairs stigma receptivity (Zhang et al., 2014). (iv) The fourth is an impairment in pollen germination, pollen tube penetration and sperm delivery to the sac. Heat stress affects the balance of ions (such as K^+^ and Ca^2+^) (Yan, Wang & Li, 2002), carbohydrate metabolism (Firon et al., 2006) and regulators (phytohormones) within pollen (Sakata et al., 2010), as well as stigma vigor (Zhang et al., 2014; Wu et al., 2019), which collectively reduce pollen germination and polarized pollen tube growth.

Notably, anther dehiscence is the initial step of pollination and is highly susceptible to heat stress; thus, anther dehiscence was suggested to be a selective marker for screening heat tolerance (Matsui & Omasa, 2002). However, we observed that there are few pollen grains inside the anthers under normal temperature (Fig. 2A) and plenty of pollen grains inside the anthers under heat condition (Fig. 2B), this observation indicate that heat stress prevents the majority of pollen grains from escaping dehisced anthers, thus reducing the number of pollen grains available for successful fertilization. This result may explain why the percentage of dehisced thecae (indicated by a basal or apical slit or aperture) was not always strongly correlated with spikelet fertility under heat stress conditions in Kobayashi et al. (2011) research. Thus, simply screening for slits or apertures in the anthers cannot ensure successful pollination will occur in heat-stressed plants. We propose that dehisced anthers (characterized by slits or apertures) with low levels of residual pollen grains in the thecae, are guarantees for effective pollination.

**Figure 3:**
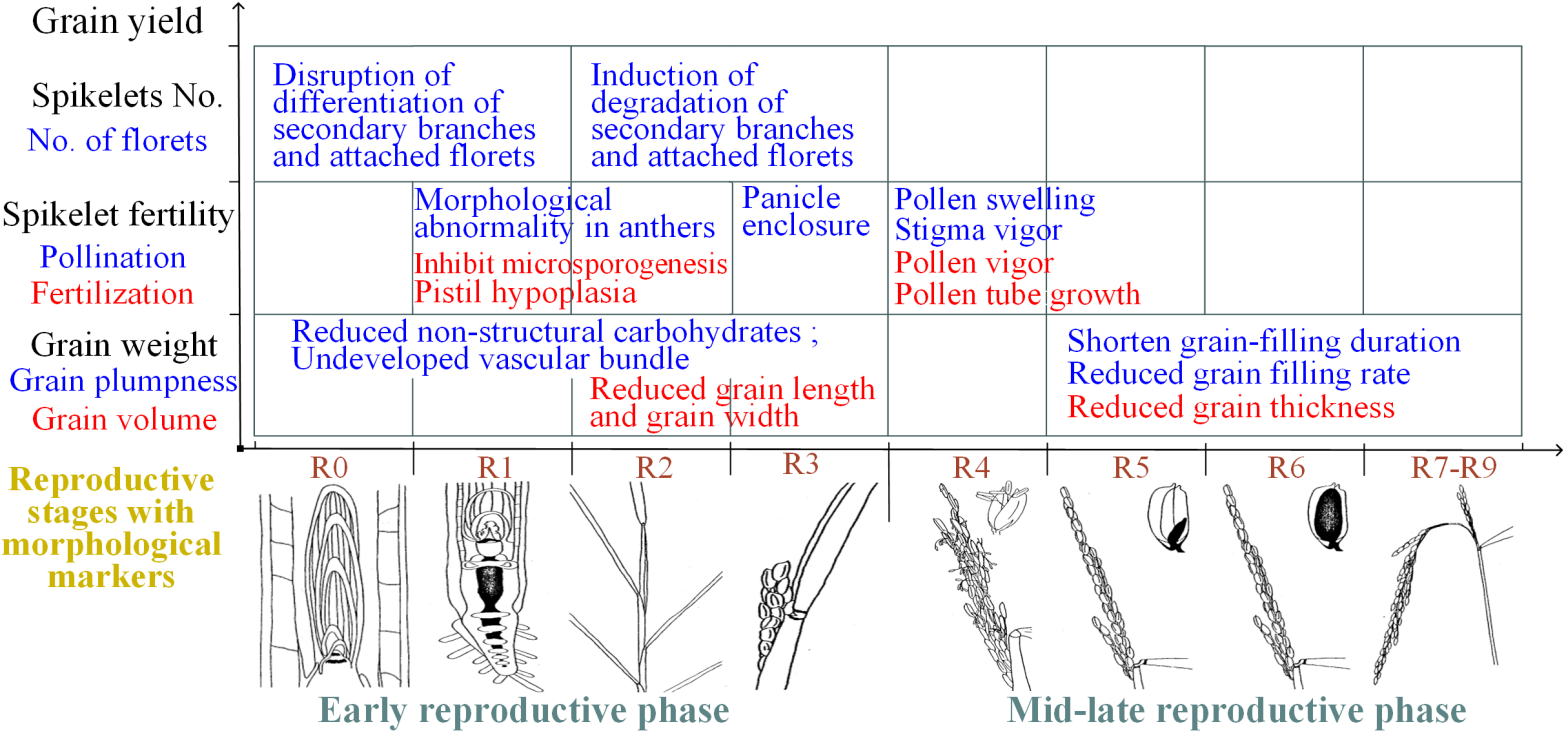
Summary of the effects of heat on yield traits of rice during different reproductive stages. R0: panicle development has initiated; R1: panicle branches have formed; R2: the flag leaf collar has formed; R3: the panicle has emerged from the boot; R4: one or more florets on the main stem panicle has reached anthesis; R5: at least one caryopsis on the main stem panicle has elongation to the end of the hull; R6: at least one caryopsis on the main stem panicle has elongated to the end of the hull; R7: at least one grain on the main stem panicle has a yellow hull; R8: at least one grain on the main stem panicle has a brown hull; and R9: all grains that reached R6 have brown hulls. The illustrations of the reproductive stages with morphological markers are adapted from those by Counce, Keisling & Mitchell (2000).

In summary, spikelet sterility induced by heat stress can be attributed primarily to physiological abnormalities in the reproductive organs during flowering and the associated mechanisms of the effects of heat stress during the early reproductive phase differ, which is mainly attributed to structural and morphological abnormalities of reproductive organs (Fig. 3).

### Effect of heat stress on grain weight

Rice grain weight is determined by multiplying grain volume by grain plumpness. Heat stress during the reproductive phase adversely affects grain volume and grain plumpness (Wang et al., 2016b). Small grain size in heat-stressed rice plants is characterized by both reduced grain width and reduced grain length when the plants are subjected to high-temperature stress during the panicle-initiation stage (Takeoka et al., 1991; Wu et al., 2016). However, small gain size was attributed to reduced grain thickness when plants were subjected to high-temperature stress during the grain-filling stage (Mohammed, Cothren & Tarpley, 2013). Moreover, grain plumpness is associated with the duration of grain filling and the grain-filling rate. A reduction in grain plumpness induced by heat stress during the early reproductive phase was associated with a reduced content of nonstructural carbohydrates in the stems (Li, 2012) and hindered vascular bundle development (Zhang et al., 2009). However, reduced grain plumpness has also been attributed to a shortened duration of grain filling, although the grain-filling rate was shown to increase in response to moderately high temperatures during the grain-filling stage (Cao et al., 2016; Dou et al., 2017). Notably, the early termination of grain filling induced by moderately high temperatures was the result of reduced sink activity due to early panicle senescence, not a lack of assimilates (Kim et al., 2011). In conclusion, the reduced grain weight caused by heat stress during panicle initiation not only is due to reduced amounts of nonstructural carbohydrates, undeveloped vascular bundles and reduced grain length and width but also is attributed to a shortened grain-filling duration, a reduced grain-filling rate and decreased grain width when exposure to heat stress occurs during grain filling (Fig. 3).

## Responses of phytohormones to heat stress and their role in yield components

### Phytohormone changes in response to heat stress affect the number of spikelets per panicle

**Figure 4:**
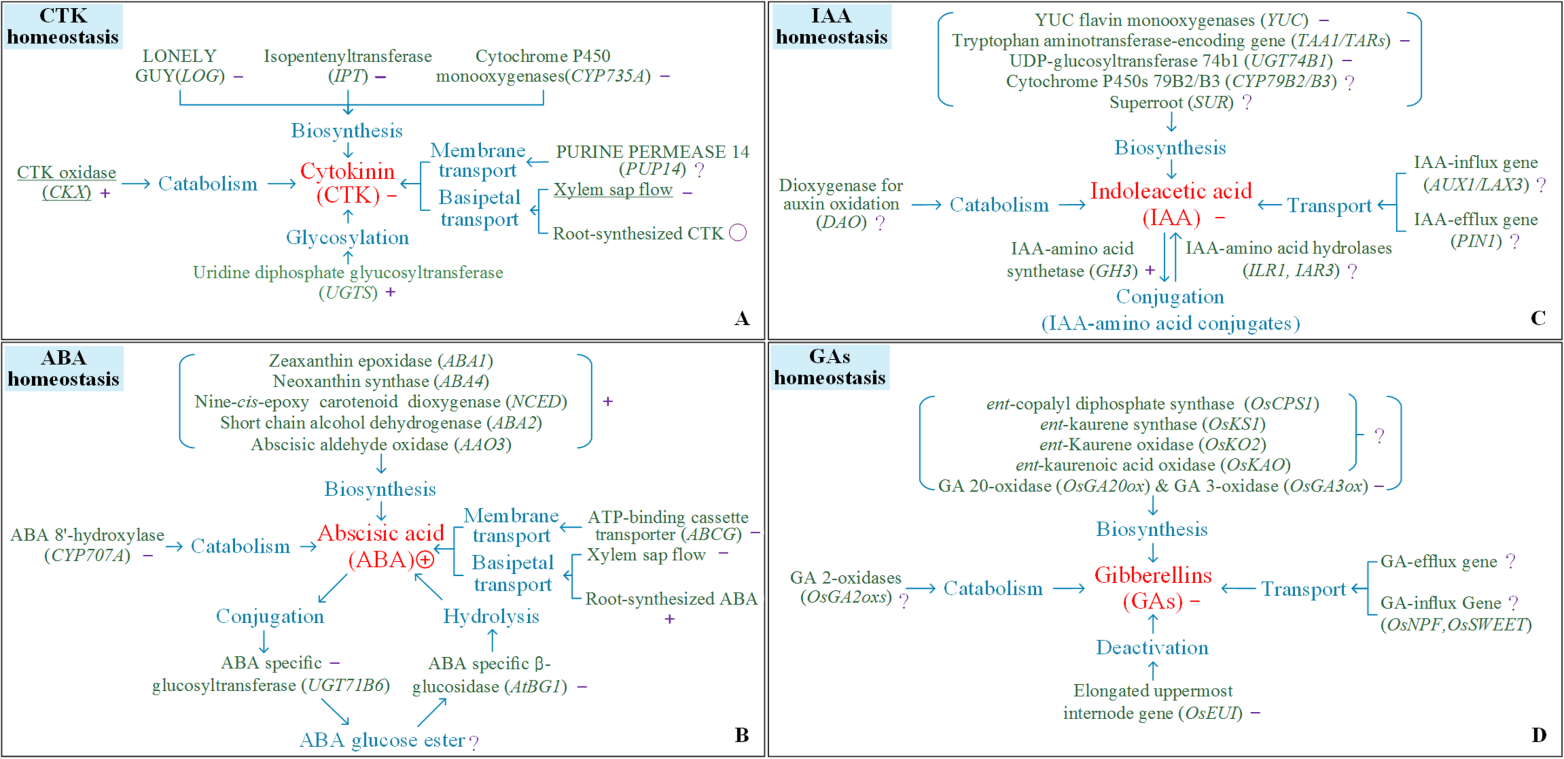
Response of processes involving the homeostasis of cytokinin, indole-3-acetic acid, abscisic acid, and gibberellin. (A) CTK homeostasis, (B) IAA homeostasis, (C) ABA homeostasis, (D) GAs homeostasis. The symbols +, −, Ο, and ? indicate an increase, a decrease, steadiness, and an undefined response, respectively, for a certain trait or process in response to heat conditions.

Heat stress reduces CTK contents. Reductions in CTK contents in the young panicles of heat-stressed rice plants are due to the following mechanisms: (i) reductions in the long-distance transport of CTKs from the roots to the shoots; (ii) inhibition of CTK biosynthesis via decreased biosynthesis of related enzymes (isopentenylation of adenosine phosphate by isopentenyltransferases (IPTs), cytochrome P450 mono-oxygenase (CYP735A) and LONELY GUY (LOG)); and (iii) increases in the catabolism of CTKs due to increased activity of CTK metabolism-related enzymes (cytokinin oxidase/dehydrogenase (CKX)) and CTK glycosylation (Skalák et al., 2016; Tripathi et al., 2012; Vyroubalová et al., 2009; Wu et al., 2017, 2016). Additional studies are necessary to understand how the membrane transport of CTKs is involved in the response to heat stress (Fig. 4A).

The number of spikelets per panicle is affected mainly by heat stress during panicle initiation (Fig. 3). Changes in CTK contents and CTK homeostasis in young panicles regulate the number of spikelets per panicle in rice (Ha et al., 2012). Under heat stress conditions, heat-susceptible varieties presented significant reductions in panicle CTKs accompanied by reduced numbers of spikelets per panicle in response to heat stress, while a heat-tolerant variety presented stable levels of panicle CTKs and a relatively more numbers of spikelets per panicle in response to heat stress. Changes in panicle CTKs correlated with a decrease in the number of spikelets per panicle (Wu et al., 2016). We determined that increased transport of root-derived CTKs and stable CKX activity within panicles are the two key processes that play a vital role in maintaining panicle size in rice varieties (Wu et al., 2017). In Arabidopsis, reduced degradation, stable local biosynthesis, and/or increased translocation of CTKs from the roots likely contributed to the stability of CTKs in target organs and facilitated adaptations to abiotic stress (Ha et al., 2012). Genetic manipulation of CTK translocation and degradation would be useful to stabilize panicle size in rice under heat stress.

### Phytohormone changes affect spikelet fertility under heat stress

Spikelet fertility is affected by heat stress during panicle initiation and flowering (Fig. 3). In rice, reduced levels of IAA in young panicles are closely related to decreased spikelet fertility, anther dehiscence and pollen vigor (Wu et al., 2016). In barley and Arabidopsis, reduced levels of IAA in the anthers were shown to be attributed to reduced expression of the IAA biosynthesis gene *YUCCA*, leading to anther indehiscence and reduced pollen vigor that could be rescued by applications of exogenous IAA (Sakata et al., 2010). However, in cotton plants, high levels of background IAA in anthers weakened the defense response of the anthers to heat stress (Min et al., 2014). It is speculated that excessively low or high levels of IAA due to disturbances in IAA homeostasis during the heat stress response hinder anther dehiscence. It was recently reported that heat stress during panicle initiation reduced spikelet fertility by inducing panicle enclosure, which can be attributed mainly to deficiencies in GA_1_ and IAA (Wu et al., 2016). IAA regulates panicle expansion by regulating GA_1_ biosynthesis (Yin et al., 2007). Thus, we speculate that panicle enclosure in heat-stressed plants may be attributed to reduced GA_1_ biosynthesis due to decrease in IAA induced by heat stress.

Heat stress suppresses the biosynthesis of IAA by downregulating the expression of genes involved in IAA biosynthesis, including tryptophan aminotransferase-encoding genes (*TAA1/TAR*s), YUC flavin monooxygenase (*YUC*) genes, and UDP-glucosyltransferase 74b1 (*UGT74B1*) (Guo et al., 2016; Sharma et al., 2018), and reduces the amount of IAA by upregulating the expression of the IAA-amino acid synthetase gene (*GH3*) in rice (Du et al., 2012), which positively regulates the formation of IAA-amino acid conjugates (Mittag, Gabrielyan & Ludwig-Müller, 2015). However, information on the response of genes involved in the degradation of IAA-amino acid conjugates (*ILR1*, *IAR3*) and the transport of IAA (*AUX1*, *LAX3*, *PIN1*) during heat stress in rice is limited. Monitoring isotope-labelled ^14^C-IAA in root tissues of pea revealed that heat stress reduced IAA transport; however, the sensitivity of the diffusion component (the IAA-influx/efflux gene) to temperature and IAA concentration has not been determined (Gladish, Sutter & Rost, 2000). In summary, heat stress suppresses the biosynthesis of IAA and promotes the formation of IAA-amino acid conjugates, yet information on the effects of heat on the catabolism and transport of IAA in rice plants is limited (Fig. 4C).

Heat stress reduces the content of GAs in young panicles of rice varieties (Wu et al., 2016). The GA biosynthetic pathway is catalyzed by several enzymes. In Arabidopsis, the GA 20-oxidase genes (*GA20ox1*, *GA20ox2*, *GA20ox3*) and GA3-oxidase genes (*GA3ox1*, *GA3ox2*), which regulate the late steps of the GA biosynthetic pathway, are suppressed by heat stress (Toh et al., 2008). However, the genes that regulate the early steps of the GA biosynthetic pathway, including those that encode ent-copalyl diphosphate synthase (*OsCPS1*), ent-kaurene synthase (*OsKS1*), ent-Kaurene oxidase (*OsKO2*) and ent-kaurenoic acid oxidase (*OsKAO*), have seldom been investigated in plants under heat stress conditions. Information concerning the responses of the catabolism-related genes (*GA 2-oxidases*, *OsGA2oxs*) and transport-related genes (*OsNPF* and *OsSWEET*) to heat stress is also limited. According to existing results, heat stress suppresses the biosynthesis and promotes the deactivation of GA, but few studies have documented the effects of heat on the catabolism and transport of GA in rice plants (Fig. 4D).

The major cause of spikelet sterility in rice is heat stress-mediated decline in endogenous IAA levels in anthers, which is associated with the expression of IAA biosynthesis (*YUC*) and transport genes (*AUX*, *PIN*) in rice under high temperature (Sharma et al., 2018). In barley and Arabidopsis, the *YUC* gene was shown to affect spikelet fertility by regulating male sterility under heat stress (Sakata et al., 2010). GA is also a key phytohormone involved in spikelet fertility, but the molecular genetic and biochemical mechanisms of GA in male fertility remain largely unknown (Kwon & Paek, 2016). In thermo-sensitive genic male-sterile lines, heat stress suppressed the expression of the *EUI* gene, which induced the deactivation of GA (Xiao et al., 2005). Additional studies on the role of GA homeostasis in spikelet fertility in plants under heat stress are needed.

### Phytohormone changes affect grain weight under heat stress

Grain weight is impaired by heat stress during panicle initiation and grain filling (Fig. 3). Most of the related previous studies have explored the physiological mechanisms of heat stress on grain weight during grain filling, revealing the regulatory role of IAA, GA, ABA and CTK on grain weight under heat stress during grain filling (Wu et al., 2016); the results are summarized here. (i) From a physiological perspective, grain weight is determined by multiplying the endosperm number by the endosperm weight, which is regulated by phytohormones such as CTK, ABA and IAA (Javid et al., 2011; Nayar et al., 2013). Changes in endosperm CTK, IAA and ABA levels induced by heat stress lead to reduced grain weight in rice varieties (Cao et al., 2016). In wheat, changes in grain weight are accompanied by changes in grain CTK content under heat stress, but exogenous CTK can increase the final grain weight under heat conditions (Banowetz, Ammar & Chen, 1999). (ii) From an agronomic perspective, grain weight is determined by multiplying the grain-filling rate by the duration of grain filling; these metrics are regulated by phytohormones during heat stress, as summarized here. (1) In terms of the grain-filling rate, changes are closely associated with the response of phytohormones such as ABA and GA (Liu et al., 2007). (2) In terms of the duration of grain filling, early panicle senescence in heat stress-stressed plants is the main cause of relatively short periods of grain filling (Kim et al., 2011). However, stay-green traits mitigate the negative impacts of heat stress on the grain because of an extended period of grain filling in rice (Kobata et al., 2015).

ABA, which acts as an important mobile signal, is synthesized in the roots and translocated to target organs via the xylem and phloem (Kuromori et al., 2010). In rice plants, heat stress was shown to have no overt effects on the ABA concentration in the roots but increased the amount of continuous xylem sap flow for at least two weeks (Wu et al., 2017). Our previous data (Wu et al., 2016) indicated that changes in ABA transportation in rice were closely correlated with the effects of heat on panicle ABA contents (*r* = 0.66, *P* < 0.05, *n* = 12). In aerial organs, heat exposure increased the ABA content by upregulating ABA biosynthesis-related genes, including the zeaxanthin epoxidase genes (*ABA1*, *ABA2*, *ABA4*, *AAO3*) and the 9-cis-epoxycarotenoid dioxygenase genes (*NCED2*, *NCED5*, *NCED9*), but downregulated expression of the ABA 8′-hydroxylase gene *CYP707A* (Toh et al., 2008), the glucosyltransferase gene *UGT75B1*, and the glucosidase gene *AtBG1* (Dobrá et al., 2015). Figure 4B illustrates the processes that are involved in ABA homeostasis and that respond to heat stress. However, little is known about the key processes that determine the ABA content in target organs under heat stress. ABA transport and the processes involved in ABA metabolism should be studied in parallel to understand the response of local ABA contents to heat stress.

## Conclusion and future outlook

The yield traits of rice respond differently to heat stress at different reproductive stages, the results of which are summarized here. (i) The number of spikelets per panicle is reduced by heat stress during panicle initiation but is not affected by heat stress during flowering and grain filling. In addition, the reduction in the number of spikelets per panicle in heat-stressed plants is due to the attenuated differentiation of secondary branches and their attached florets as well as the promotion of their degradation during panicle-initiation stage. (ii) Spikelet sterility induced by heat stress can be attributed primarily to physiological abnormalities in the reproductive organs during the flowering stage but also can be attributed to structural and morphological abnormalities in reproductive organs during the panicle-initiation stage. (iii) Last, the relatively low grain weight caused by heat stress during panicle initiation is due to a reduction in nonstructural carbohydrates, undeveloped vascular bundles and a reduction in grain length and width, while a shortened grain-filling duration, reduced grain-filling rate and decreased grain width affect grain weight when heat stress occurs during the grain-filling stage. Different responses of yield traits to heat stress have been identified in rice during the panicle-initiation, flowering and grain-filling stages. Although many heat-tolerant rice varieties have been identified, most of these varieties endure heat injury only during specific reproductive stages, mainly during flowering. To cope with unpredictable heat events in the future, we should screen and breed rice varieties with comprehensive tolerance to heat stress throughout the entire reproductive phase.

The physiological and molecular mechanisms of the effects of heat on rice grain yield have drawn much attention. Under heat conditions, CTK and ABA modulate the number of spikelets per panicle; IAA and GA are thought to be involved in spikelet fertility; and IAA, GA, ABA and CTK mediate grain weight. The responses of yield traits to heat stress are regulated by phytohormone levels, which are determined by phytohormone homeostasis. Currently, biosynthesis and transport are purported to be the key processes that determine phytohormone levels in and final grain yields of rice under heat stress. Studies on phytohormone homeostatic responses are needed to further reveal the key processes that determine phytohormone levels under heat conditions.

## Additional Information and Declarations

### Data Availability

The following information was supplied regarding data availability: There is no raw data or code in this review article.
